# The Ras-Erk-ETS-Signaling Pathway Is a Drug Target for Longevity

**DOI:** 10.1016/j.cell.2015.06.023

**Published:** 2015-07-02

**Authors:** Cathy Slack, Nazif Alic, Andrea Foley, Melissa Cabecinha, Matthew P. Hoddinott, Linda Partridge

**Affiliations:** 1 Institute of Healthy Ageing, Department of Genetics, Evolution, and Environment, University College London, Darwin Building, Gower Street, London WC1E 6BT, UK; 2 Max Planck Institute for Biology of Ageing, Joseph-Stelzmann-Strasse 9b, 50931 Cologne, Germany

## Abstract

Identifying the molecular mechanisms that underlie aging and their pharmacological manipulation are key aims for improving lifelong human health. Here, we identify a critical role for Ras-Erk-ETS signaling in aging in *Drosophila*. We show that inhibition of Ras is sufficient for lifespan extension downstream of reduced insulin/IGF-1 (IIS) signaling. Moreover, direct reduction of Ras or Erk activity leads to increased lifespan. We identify the E-twenty six (ETS) transcriptional repressor, Anterior open (Aop), as central to lifespan extension caused by reduced IIS or Ras attenuation. Importantly, we demonstrate that adult-onset administration of the drug trametinib, a highly specific inhibitor of Ras-Erk-ETS signaling, can extend lifespan. This discovery of the Ras-Erk-ETS pathway as a pharmacological target for animal aging, together with the high degree of evolutionary conservation of the pathway, suggests that inhibition of Ras-Erk-ETS signaling may provide an effective target for anti-aging interventions in mammals.

**Video Abstract:**

## Highlights

• Reduced insulin/IGF-1 (IIS) signaling involves Ras inhibition for longevity
• Attenuation of Ras-Erk signaling extends lifespan via the Aop transcription factor
• Treatment with trametinib, an inhibitor of Ras-Erk signaling, extends lifespan
• Ras-Erk-ETS signaling may provide targets for anti-aging interventions in mammals

## Graphical Abstract

**Figure:**
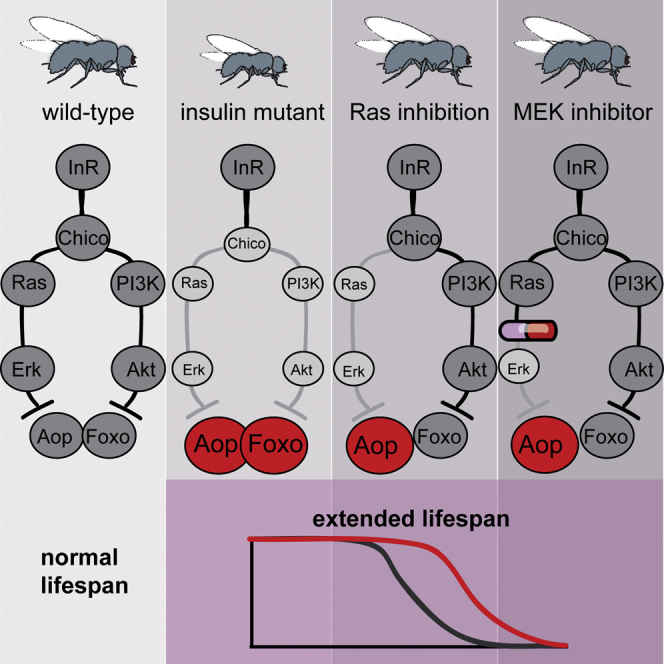

## Teaser

Ras inhibition is implicated in the longevity that arises from reduced insulin/IGF-1 signaling. In adult flies, pharmacological inhibition of Ras signaling using the Mek kinase inhibitor, trametinib, extends lifespan, revealing a new potential target for midlife anti-aging interventions.

## Introduction

Ras proteins are members of a superfamily of small GTPases that transmit signals from cell-surface receptor tyrosine kinases (RTKs) to activate multiple downstream cell signaling pathways (Schlessinger, 2000). Ras proteins thereby occupy a key position in the signaling network controlling numerous cellular processes, including proliferation, differentiation, apoptosis, senescence, and metabolism (Goitre et al., 2014). Hyper-activation of Ras is highly oncogenic, and approximately one-third of human tumors present with *ras* mutation, a context in which Ras has been extensively studied (Stephen et al., 2014).

Ras proteins are molecular switches that cycle between an inactive GDP-bound state and an active GTP-bound state, the balance of which is determined by the competing activities of guanine nucleotide exchange factors (GEFs) and GTPase activating proteins (GAPs) (Goitre et al., 2014; Stephen et al., 2014). In its active, GTP-bound conformation, Ras has high affinity for numerous downstream effectors of RTK signal transduction pathways, including Raf, thereby activating the extracellular signal-regulated kinase (Erk)/mitogen-activated protein kinase (Mapk)-signaling cascade and the p110 catalytic subunit of the class 1 phosphatidylinositol 3-kinase (PI3K), leading to activation of the PI3K-Akt-signaling cascade (Goitre et al., 2014; Stephen et al., 2014).

Deletion of *RAS2*, one of two Ras paralogs in the budding yeast, has long been known to extend chronological lifespan (Fabrizio et al., 2003). Recent experiments in mice indicate that this pro-longevity function of Ras inhibition may be conserved. Mice deficient in *RasGrf1*, which encodes a tissue-specific guanine exchange factor for Ras (Fernández-Medarde and Santos, 2011), have been reported to live longer and maintain a better level of motor coordination in old age than their wild-type littermates (Borrás et al., 2011). However, involvement of Ras in determining metazoan lifespan awaits direct confirmation, as RasGrf1 has affinity for several other ligands, including Rac, Rho, microtubules, PI[4,5]P2, and fasfatidic acid (Mirisola and Longo, 2011).

In mammals, Ras is a well-established signaling intermediary of the insulin/IGF-1-signaling (IIS) pathway (White, 1997), which plays an evolutionarily conserved role in the modulation of animal lifespan (Fontana et al., 2010; Kenyon, 2011; López-Otín et al., 2013). Central to the capacity of reduced IIS to extend lifespan is the regulation of the Forkhead box O (Foxo) transcription factor via the PI3K-Akt-Foxo branch (Fontana et al., 2010; Ortega-Molina et al., 2012; Slack et al., 2011), yet there are no reported pharmacological inhibitors that target this signaling pathway to increase lifespan in any species. A role for Ras downstream of IIS in the control of aging has remained largely unexplored despite its potential to offer novel molecular targets for anti-aging therapeutics.

In this study, we examine the role of Ras and its downstream signaling effectors, Erk, and the E-twenty six (ETS) transcription factors (TFs) during aging in *Drosophila*. We report that attenuation of Ras-Erk-ETS signaling is implicated in the effects of reduced IIS on aging and show that direct inhibition of Ras-Erk-ETS signaling is sufficient to extend lifespan. Importantly, we identify trametinib, a potent small-molecule inhibitor of Ras-Erk-ETS signaling, as an anti-aging intervention. These findings thereby define the inhibition of Ras-Erk-ETS signaling as an important pro-longevity mechanism that can be pharmacologically manipulated to extend animal lifespan.

## Results

### Ras Functions Downstream of IIS in Lifespan Regulation

The insulin receptor substrates (IRS) couple insulin receptor stimulation to the activation of downstream signaling pathways. By binding to Grb2/Drk protein—which in turn acts as an adaptor for the Ras GEF, SOS—IRS proteins recruit activated Ras to the activated insulin receptor.

**Figure 1:**
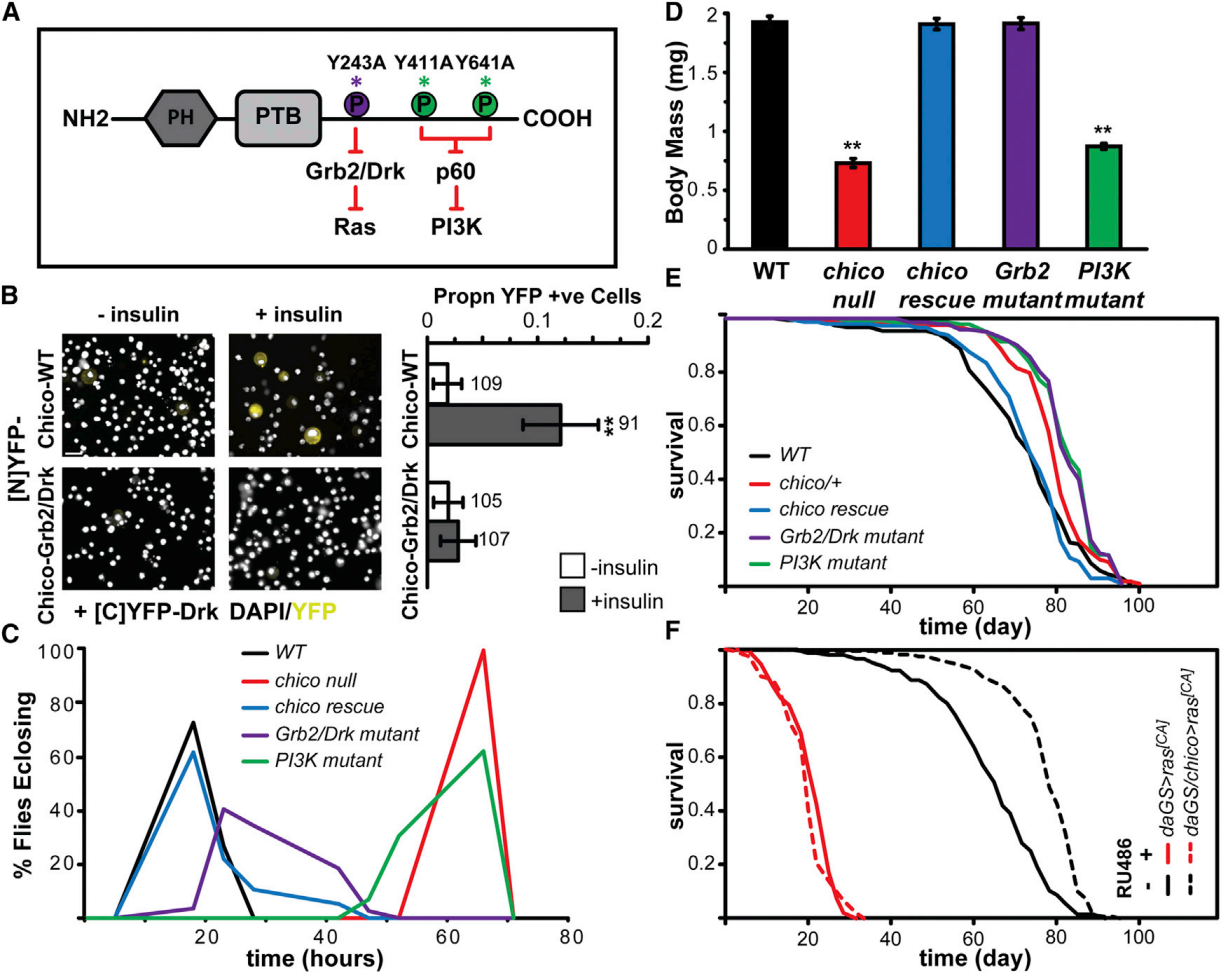
Ras Inhibition Functions Downstream of IIS to Extend Lifespan (A) Chico protein sequence with amino acid substitutions used to generate the Chico-Grb2/Drk- and Chico-PI3K-binding site mutants. (B) BiFC in S2 cells co-expressing the indicated Chico constructs with the *Drosophila* Drk protein. Proportion of YFP-positive cells ± SE; n numbers are indicated above each bar; ^∗∗^p < 0.005 Chi-square test to no insulin control. Scale bar, 10 μm. (C) Egg-to-adult development time of the indicated genotypes. See also Figure S1. (D) Fresh weight of adult females of the indicated genotypes. Mean body mass (n = 10 for each genotype) ± SEM, Anova, p < 0.0001, ^∗∗^p < 0.05 t test (compared to WT). (E) Survival of wild-type and *chico/+* heterozygous females carrying the indicated *chico* genomic rescue constructs. *chico/+* flies were long-lived compared to *WT* (p = 0.0006), which was rescued by the *chico* rescue construct (p = 0.58). Both the Grb2/Drk mutant and the PI3K mutant failed to rescue the longevity of *chico/+* flies (compared to WT construct, p = 8.36 × 10^−10^ and p = 1.32 × 10^−9^, respectively). See Table S1A. (F) Expression of constitutively active Ras blocks the beneficial effects of *chico* mutation on survival. *daGS/chico > ras*^*[CA]*^ flies show increased lifespan compared to *daGS > ras*^*[CA]*^ in the absence of RU486 (p = 3.07 × 10^−18^), but not in the presence of RU486 (p = 0.18). See Table S1B.

In order to analyze the contribution of Ras-Erk signaling to IIS-dependent lifespan extension in *Drosophila*, we mutated the proposed binding site for Grb2/Drk in the single *Drosophila* IRS protein, Chico (Figure 1A). We first confirmed that this mutation disrupts the interaction between the *Drosophila* Chico and Grb2/Drk proteins upon insulin stimulation in vivo using the bimolecular fluorescence complementation (BiFC) assay in cultured S2 cells. Wild-type (Chico-WT) and Grb2/Drk-binding mutant forms of Chico (Chico-Grb2/Drk) were tagged at their C termini with the N-terminal fragment of YFP ([N]YFP), and *Drosophila* Drk was tagged with the C-terminal fragment of YFP ([C]YFP-Drk). Each of the YFP fragments is non-fluorescent, but an interaction between the proteins of interest brings them in close proximity, allowing YFP to reform and emit a fluorescent signal. In the absence of insulin, co-expression of Chico-WT-[N]YFP or Chico-Grb2/Drk-[N]YFP with [C]YFP-Drk did not result in significant YFP fluorescence (Figure 1B). Insulin stimulation of cells co-expressing Chico-WT-[N]YFP with [C]YFP-Drk produced strong YFP fluorescence (Figure 1B, effect of insulin, p = 0.003), but not in cells co-expressing Chico-Grb2/Drk-[N]YFP with [C]YFP-Drk (Figure 1B, effect of insulin, p = 0.67). Thus, mutation of the Grb2/Drk-binding site in *Drosophila* Chico prevented its direct interaction with Drk in vivo.

**Figure S1:**
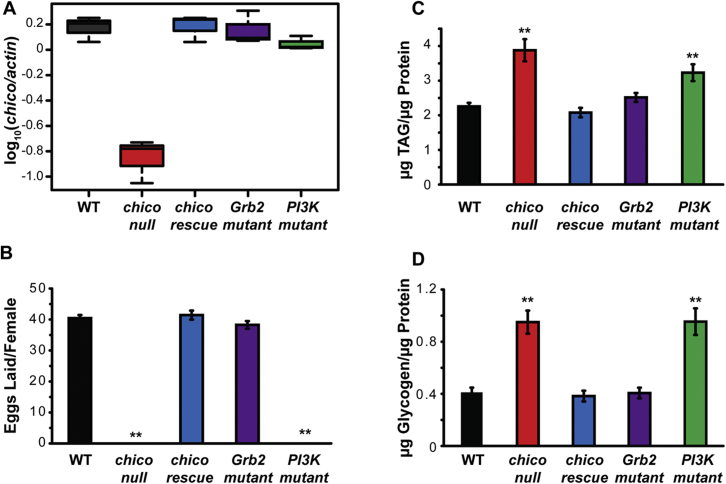
Expression Levels and Developmental Phenotypes of *chico* Genomic Rescue Constructs, Related to Figure 1 (A) Log-transformed levels of *chico* mRNA relative to *actin* in females of the indicated genotypes. *chico* expression was significantly reduced in *chico* mutants (p < 0.05, t test compared to WT controls), but not in the presence of any of the genomic rescue constructs. Means ± SEM. (B–D) The PI3K-binding site, but not the Grb2/Drk-binding site, of Chico is essential for female sterility (B) and increased metabolic stores (C and D). Means ± SEM, ^∗∗^p < 0.05, t test (compared to WT controls).

To examine the role of the Grb2/Drk-binding site in fly physiology, we generated flies carrying this mutation as a genomic rescue construct alongside genomic rescue constructs containing the wild-type *chico* sequence as well as a second construct with mutations to disrupt binding of Chico to the p60 subunit of PI3K (Figure 1A). All constructs included the *cis*-regulatory sequences to allow *chico* expression in its normal spatial and temporal pattern and were inserted into the same genomic location, producing similar levels of *chico* mRNA expression (Figure S1A).We were therefore able to assess the ability of wild-type or mutant forms of Chico to complement the phenotypes of *chico* loss-of-function mutants under equivalent physiological conditions.

To validate our experimental strategy, we examined the previously characterized role of the different domains of Chico in cell proliferation and growth (Oldham et al., 2002). We confirmed that the wild-type *chico* genomic rescue construct fully restored several phenotypic defects associated with *chico* null mutation, including developmental delay, reduced growth (Figures 1C and 1D), female sterility, and increased glycogen and lipid storage (Figures S1B–S1D). The Grb2/Drk-binding site mutant also fully rescued these phenotypes to the same extent as the wild-type rescue construct, confirming that the presence of a functional Grb2/Drk-binding site is not required for these functions of Chico (Figures 1C, 1D, and S1B–S1D). The PI3K-binding mutant behaved similarly to complete loss of *chico* function (Figures 1C, 1D, and S1B–S1D), confirming that Chico requires PI3K docking sites for its wild-type function in growth and metabolism (Oldham et al., 2002), thereby substantiating our genetic approach.

We then examined the ability of our genomic rescue constructs to rescue the lifespan extension associated with *chico* mutation. To circumvent confounding effects of differences in body size, metabolism, and fertility, we performed the lifespan experiments in a *chico* heterozygous background, where these phenotypes are not obvious (Oldham et al., 2002). *chico* heterozygotes were long-lived compared to wild-type controls (Figure 1E, median lifespan +12%, p = 0.0006). As expected, the wild-type *chico* construct was able to fully restore the lifespan of *chico/+* to that of wild-type flies (Figure 1E, p = 0.58). In contrast, the PI3K-binding mutant failed to rescue *chico* lifespan extension (Figure 1E, p = 1.32 × 10^−9^). Interestingly, the Grb2/Drk-binding mutant also failed to rescue the *chico* lifespan extension (Figure 1E, p = 8.36 × 10^−10^), and both the PI3K-binding and Grb2/Drk-binding mutants showed an increase in median lifespan of 15% compared to the wild-type control. To compare the extent of lifespan extension between the two mutants, we used Cox proportional hazards (CPH) analysis with relevant a priori contrasts: the lifespan extension observed in the Grb2/Drk-binding mutant was not significantly different from that in the PI3K-binding mutant (p = 0.98, Table S1A). Thus, inhibition of signaling from Chico to Ras was sufficient to extend lifespan and to the same degree as inhibition of signaling from Chico to PI3K.

**Figure S2:**
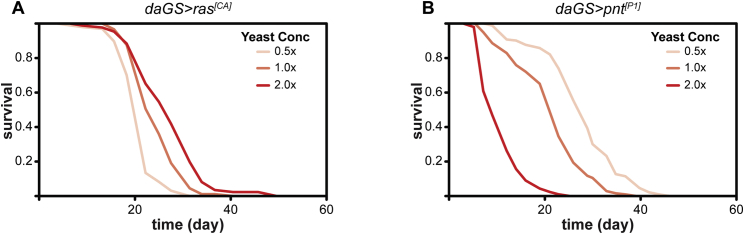
Effects of Yeast Concentration on the Survival of Flies Expressing Constitutively Active Ras or Constitutively Active Pointed, Related to Figures 1 and 4 (A) Survival of *daGS > ras*^*[CA]*^ flies in the presence of RU486 under different dietary yeast concentrations. Log-rank detected a significant increase in survival between 0.5x and 1.0x yeast concentrations (increase in median lifespan of 13%, p = 6.28x10^−8^) and between 1.0x and 2.0x yeast concentrations (increase in median lifespan of 11%, p = 7.41x10^−4^). 0.5x yeast: n = 97 deaths/0 censors, median lifespan = 16 days; 1.0x: n = 89/0, median lifespan = 18 days; 2.0x: n = 86/0, median lifespan = 20 days. (B) Survival of *daGS > pnt*^*[P1]*^ flies in the presence of RU486 under different dietary yeast concentrations. Log-rank detected a significant increase in survival between 1.0x and 0.5x yeast concentrations (increase in median lifespan of 40%, p = 4.68x10^−14^) and between 2.0x and 1.0x yeast concentrations (increase in median lifespan of 150%, p = 1.62x10^−37^). 0.5x yeast: n = 163 deaths/0 censors, median lifespan = 28 days; 1.0x: n = 159/0, median lifespan = 20 days; 2.0x: n = 152/0, median lifespan = 8 days.

Having established that the presence of a functional Grb2/Drk-binding site in Chico is required for its wild-type function in lifespan, we tested whether ectopic activation of Ras can block the beneficial effects of *chico* mutation on lifespan. We expressed a constitutively active form of *Drosophila* Ras (*ras*^*[CA]*^) under the control of the inducible, ubiquitous *daGS* driver in flies either wild-type or mutant for *chico*. In non-induced flies, mutation of *chico* resulted in a significant increase in lifespan (Figure 1F, 18% median extension, p = 3.07 × 10^−18^). Ubiquitous expression of *ras*^*[CA]*^ in adults using RU486 gave short-lived flies (Figure 1F, p = 3.97 × 10^−67^), but altering the concentration of dietary yeast improved their survival; thus, their lifespan retained plasticity (Figure S2A). Nevertheless, mutation of *chico* did not increase their lifespan (Figure 1F, p = 0.18). CPH confirmed that the presence of the *chico* mutation had a significantly different impact on the survival of flies with induced *ras*^*[CA]*^ compared to the non-induced controls (p = 7.10 × 10^−11^, Table S1B). Hence, activation of Ras is sufficient to prevent any beneficial effects of *chico* mutation on survival.

### Direct Inhibition of Ras-Erk Signaling Extends Lifespan

**Figure 2:**
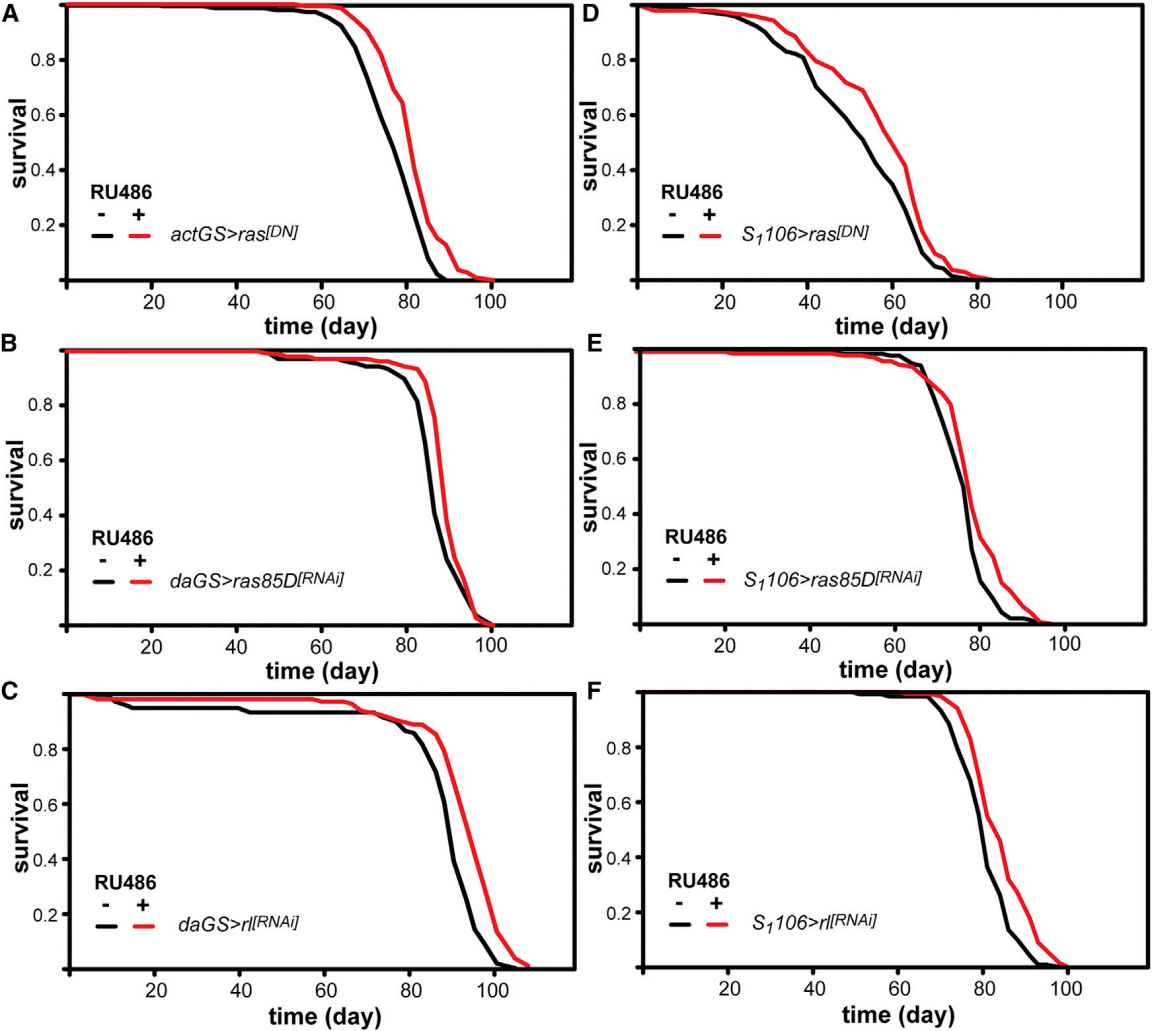
Direct Inhibition of Ras-Erk Signaling Extends Lifespan (A) Ubiquitous adult-onset expression of *ras*^*[DN]*^ increases lifespan (p = 1.26 × 10^−7^). See Table S2A. (B) Ubiquitous adult onset of *ras85D* knockdown by RNAi increases lifespan (p = 2 × 10^−3^). See Table S2B. (C) Ubiquitous adult-onset knockdown of *rl* expression by RNAi increases lifespan (p = 1.46 × 10^−7^). See Table S2C. (D) Adult gut/fat body expression of *ras*^*[DN]*^ extends lifespan (p = 3 × 10^−3^). See Table S2D. (E) Adult gut/fat body knockdown of *ras85D* expression by RNAi extends lifespan (p = 4 × 10^−4^). See Table S2E. (F) Adult gut/fat body knockdown of *rl* expression by RNAi extends lifespan (p = 10^−5^). See Table S2F.

We next tested whether direct inhibition of Ras itself is sufficient to extend lifespan by expressing a dominant-negative form of human Ras (*ras*^*[DN]*^). Adult-onset, ubiquitous expression of *ras*^*[DN]*^ resulted in a modest but significant lifespan extension (Figure 2A, median survival +8%, p = 1.26 × 10^−7^). In addition, we examined the effects on lifespan of RNAi-mediated knockdown of expression of the *Drosophila ras85D* gene (*ras85D*^*[RNAi]*^). Adult-onset, ubiquitous RNAi against *ras85D* also resulted in a significant increase in lifespan (Figure 2B, median survival +4%, p = 0.002), confirming that reducing Ras activity is sufficient to extend lifespan.

The canonical output of Ras activation is the Erk/Mapk pathway (Stephen et al., 2014). To determine whether a reduction in Erk activity is sufficient to extend lifespan, we used RNAi to knock down expression of the *Drosophila* ortholog of *Erk*, *rolled* (*rl*^*[RNAi]*^). Adult-onset, ubiquitous RNAi knockdown of *rl* significantly increased lifespan (Figure 2C, median survival +6%, p = 1.46 × 10^−7^). Taken together, these data demonstrate that direct inhibition of Ras-Erk signaling can extend lifespan.

The *Drosophila* gut and fat body, the latter functionally equivalent to mammalian liver and adipose, have an evolutionarily conserved function in aging (Fontana et al., 2010). We therefore examined whether confining Ras-Erk inhibition to these organs is sufficient to extend lifespan. Using the inducible, gut- and fat body-specific *S*_*1*_*106* driver (Poirier et al., 2008), we expressed *ras*^*[DN]*^, *ras85D*^*[RNAi]*^, or *rl*^*[RNAi]*^ specifically within these tissues. In each case, induction of expression with RU486 resulted in significant extension of lifespan (Figures 2D–2F, p = 3 × 10^−3^, p = 4 × 10^−4^ and p = 1 × 10^−5^, median lifespan +7%, +1%, and +5%, respectively). Knockdown of *ras85D* produced smaller effects on lifespan than expression of *ras*^*[DN]*^, possibly reflecting redundancy between Ras85D and the two other *Drosophila* Ras homologs, Ras64B and Roughened, which are also expressed in the adult fat body (Chintapalli et al., 2007). Nevertheless, our data clearly show that inhibition of Ras-Erk signaling in just these two tissues is sufficient to extend lifespan.

### The ETS Transcription Factor, Aop, Mediates Ras-Dependent Lifespan Effects

**Figure 3:**
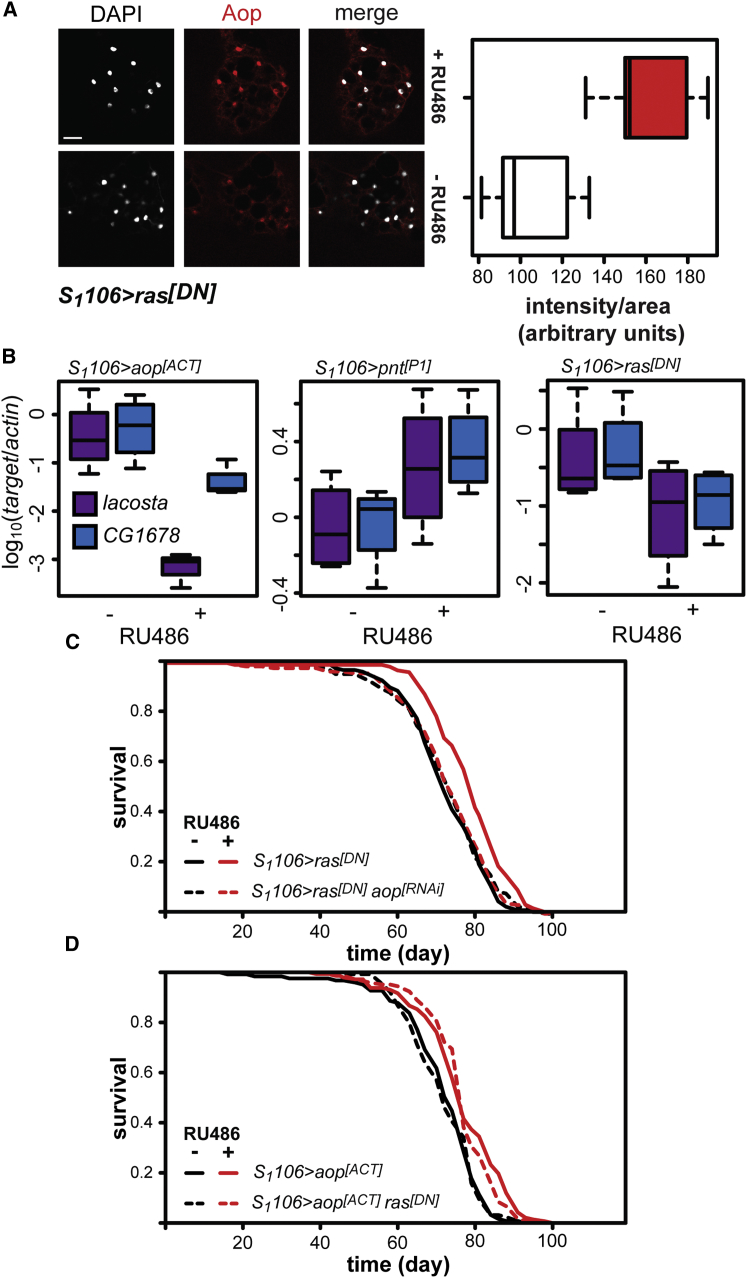
Aop Functions Downstream of Ras in the Adult Fly to Control Lifespan (A) Nuclear localization of Aop protein (red) increases in intensity in dissected adult abdominal fat bodies of *S*_*1*_*106 > ras*^*[DN]*^ females fed RU486. (n = 5, p = 5 × 10^−3^, t test). Nuclei are stained with DAPI (white). Scale bar, 25 μm. Intensity quantifications are shown as box plots. (B) Box plots of log-transformed levels of lacosta (lcs) and CG1678 mRNAs relative to actin in females of the indicated genotypes. *S*_*1*_*106 > aop*^*[ACT]*^: mixed effects linear model (MELM) (n = 3–4): significant effect of RU486 (p < 10^−4^), transcript (p = 2 × 10^−3^) and their interaction (p = 4 × 10^−3^), with significant differences between – and + RU486 for each transcript (p < 0.05, post hoc t test); *S*_*1*_*106*>*pnt*^*[P1]*^: significant effect of RU486 only (p = 0.02, n = 4, MELM); *S*_*1*_*106*>*ras*^*[DN]*^ significant effect of RU486 only (p = 0.02, n = 4, MELM). (C) *S*_*1*_*106 > ras*^*[DN]*^ show increased lifespan in the presence of RU486 (p = 2 × 10^−6^), but *S*_*1*_*106 > ras*^*[DN]*^*aop*^*[RNAi]*^ females do not (p = 0.95). See Table S3A. (D) *S*_*1*_*106 > aop*^*[ACT]*^ females show increased lifespan in the presence of RU486 (p = 2 × 10^−5^). *S*_*1*_*106 > aop*^*[ACT]*^*ras*^*[DN]*^ females also show increased lifespan in the presence of RU486 (p = 4x10^−6^) but are no longer lived than *S*_*1*_*106 > aop*^*[ACT]*^. See Table S3B.

Key outputs of the Ras-Erk-signaling pathway during *Drosophila* development are two ETS TFs: Pointed (Pnt), a transcriptional activator stimulated by the Ras-Erk pathway, and Anterior open (Aop), a transcriptional repressor inhibited by the pathway. These two TFs regulate the same genes by binding to the same regulatory elements but with opposing outcomes (Brunner et al., 1994; Halfon et al., 2000; O’Neill et al., 1994). Inhibition of Ras activity promotes the nuclear localization of Aop in *Drosophila* larvae, and we found that, similarly, adult-onset expression of *ras*^*[DN]*^ using the *S*_*1*_*106* driver resulted in a significant increase in nuclear Aop in the adult fat body (Figure 3A, p = 5 × 10^−3^).

To further assess the transcriptional activity of Aop upon Ras inhibition, we first had to identify Aop targets in the adult. An activated form of Aop (Aop^[ACT]^), mutated for eight Mapk phosphorylation sites and hence resistant to repression by Erk, shows high transcriptional activity in vivo (Brunner et al., 1994; O’Neill et al., 1994; Rebay and Rubin, 1995). Microarray analysis of gene expression in adult fat bodies identified two neighboring genes, *la costa* (*lcs*) and *CG1678*, as strongly repressed after induction of *aop*^*[ACT]*^ (Alic et al., 2014). Quantitative RT-PCR on RNA isolated from abdominal fat bodies confirmed this repression (Figure 3B, p < 10^−4^). Consistent with *lcs* and *CG1678* being targets of Aop in the adult fat body, expression of an active form of *pnt* (*pnt*^*[P1]*^) increased their transcript levels (Figure 3B, p = 0.02) while expression of *ras*^*[DN]*^ decreased their expression upon RU486 induction (Figure 3B, effect of RU486, p = 0.02). Together with the increase in nuclear localization of Aop, this strongly indicates that Ras inhibition activates Aop in the adult fat body.

We have recently shown that expression of *aop*^*[ACT]*^ in the gut and fat body of adult *Drosophila* can extend lifespan (Alic et al., 2014). We therefore examined whether Ras and Aop act in the same pathway to influence adult lifespan. We previously showed that targeted knockdown of *aop* expression specifically in the adult gut and fat body using RNAi has no significant effect on lifespan (Alic et al., 2014). RNAi-mediated knockdown of *aop* expression was, however, sufficient to completely block the lifespan extension associated with gut and fat body expression of *ras*^*[DN]*^ (Figure 3C; RU486 had a significant effect on the lifespan of *S*_*1*_*106 > ras*^*[DN]*^ flies, median lifespan +11%, p = 2 × 10^−6^, but not in *S*_*1*_*106 > ras*^*[DN]*^*aop*^*[RNAi]*^ flies, p = 0.95). CPH analysis confirmed that there was a significant difference in the response to RU486 in the two lines (RU486 by genotype interaction, p = 3.2 × 10^−3^, Table S3A). Hence, Aop is required for the beneficial effects of Ras inhibition on lifespan.

We next tested whether inhibition of Ras can increase lifespan in addition to activation of Aop in the adult gut and fat body. Inducing *ras*^*[DN]*^ expression in flies already expressing *aop*^*[ACT]*^ did not result in any further increase in lifespan (Figure 3D). Indeed, CPH detected a significant effect of RU486 (p < 10^−4^) but found no significant difference in the response to RU486 between *S*_*1*_*106 > aop*^*[ACT]*^ and *S*_*1*_*106 > aop*^*[ACT]*^*ras*^*[DN]*^ flies (RU486 by genotype interaction p = 0.35, Table S3B). Thus, inhibition of Ras does not further increase lifespan once Aop is already activated. Together with the data presented above, this suggests that Aop is both necessary and sufficient to mediate the effects of Ras inhibition on lifespan.

### AOP Is Required for *chico*-Dependent Lifespan Extension

**Figure 4:**
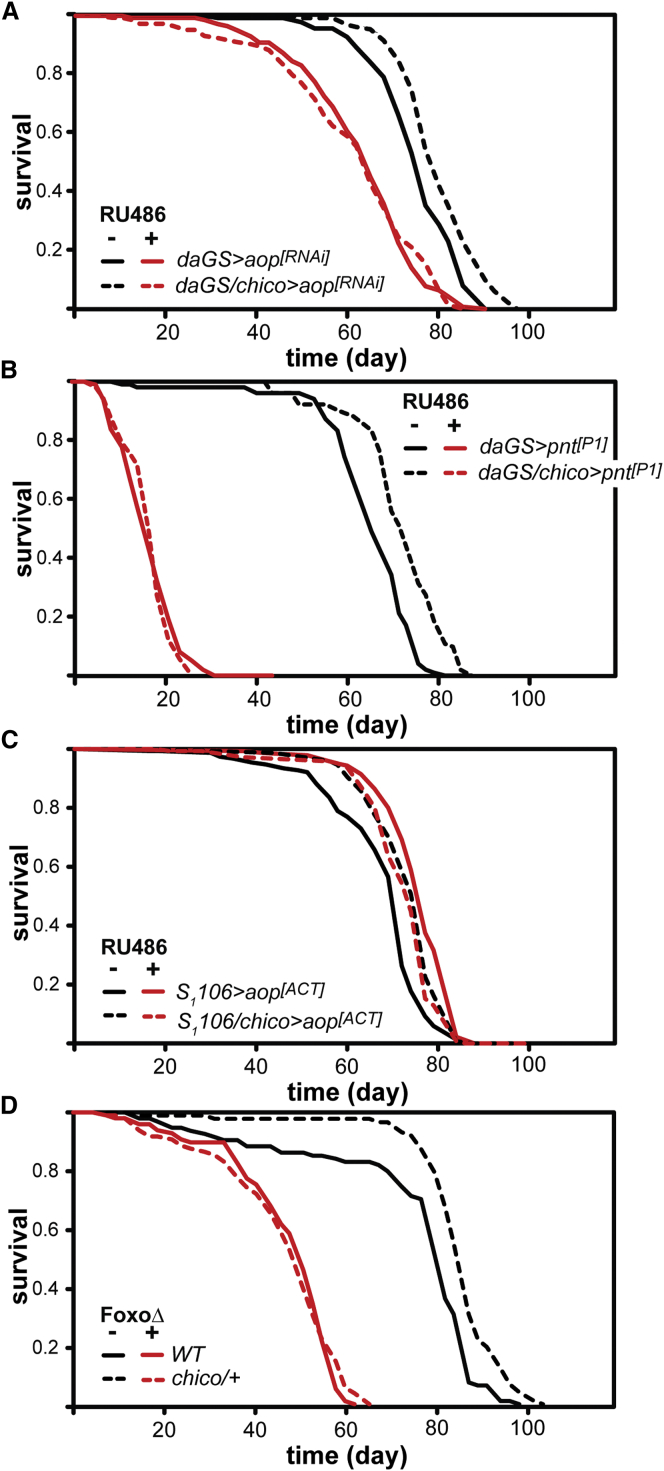
The Ras-Regulated Transcription Factor, Aop, Is Required Downstream of *chico* to Increase Survival (A) *chico* extends lifespan in *daGS > aop*^*[RNAi]*^ females in the absence of RU486 (p = 1.2 × 10^−5^), but not in the presence of RU486 (p = 0.95). See Table S4A. (B) *chico* extends lifespan in *daGS > pnt*^*[P1]*^ females in the absence of RU486 (p = 2.34 × 10^−9^), but not in the presence of RU486 (p = 0.64). See Table S4B. (C) The effects of *chico/*_*+*_ mutation and *S*_*1*_*106 > aop*^*[ACT]*^ are not additive for lifespan (*S*_*1*_*106 > aop*^*[ACT]*^ versus *S*_*1*_*106/chico > aop*^*[ACT]*^ in the RU486− condition, p = 8.84 × 10^−7^; *S*_*1*_*106 > aop*^*[ACT]*^ RU486− versus RU486+ conditions, p = 3.20 × 10^−13^; *S*_*1*_*106/chico > aop*^*[ACT]*^ RU486− versus RU486+, p = 0.29). See Table S4C. (D) *dfoxo* is required for *chico/+* lifespan extension. *chico/+* females show increased lifespan (p = 3.71 × 10^−6^), but not in the absence of *dfoxo* (p = 0.63). See Table S4D.

Our observation that impairing the ability of Chico to signal through to Ras was sufficient to extend lifespan prompted us to test whether Aop transcriptional activity also contributes to IIS-dependent lifespan extension. Ubiquitous targeted knockdown of *aop* expression by RNAi in otherwise wild-type flies significantly impaired their survival (Figure 4A, p = 2.36 × 10^−16^). Mutation of *chico* significantly increased lifespan in non-induced *daGS > aop*^*[RNAi]*^ (median lifespan +5%, p = 1.20 × 10^−5^), but not in RU486-induced flies (p = 0.95). CPH confirmed that the presence of the *chico* mutation had a significantly different impact on the lifespan of *daGS > aop*^*[RNAi]*^ flies in the presence or absence of RU486 (Table S4A, p = 3.2 × 10^−3^).

The activity of Aop counteracts that of the Pnt transcriptional activator, and hence, similarly to a reduction in Aop activity, increased Pnt activity should also block any effects of *chico* on survival. Ubiquitous expression of the constitutively active form of Pnt, *pnt*^*[P1]*^ in otherwise wild-type flies significantly impaired their survival (Figure 4B, p = 1.66 × 10^−50^). Their lifespan could not be extended by *chico* mutation (p = 0.64, Figure 4B) but remained responsive to dietary yeast (Figure S2B). At the same time, *chico* mutation significantly increased lifespan in non-induced flies (median lifespan +12%, p = 2.33 × 10^−9^). CPH analysis of the survival data confirmed that the presence of the *chico* mutation had a significantly different impact on the lifespan of *daGS > pnt*^*[P1]*^ flies in the presence or absence of RU486 (Table S4B, p = 1.4 × 10^−6^). Thus, counteracting the activity of Aop by increasing that of Pnt specifically blocked any beneficial effects of *chico* mutation on survival.

We also tested whether induction of *aop*^*[ACT]*^ in the gut and fat body could increase lifespan in addition to *chico* mutation. Each intervention alone produced a significant increase in median lifespan of 7% (Figure 4C, *aop*^*[ACT]*^ expression, p = 3.20 × 10^−13^; *chico* mutation, p = 8.84 × 10^−7^). However, the effects of each intervention on lifespan extension were less than additive (Figure 4C and Table S4C). We also confirmed that *dfoxo* is required for the lifespan extension of *chico* heterozygous mutation (Figure 4D; see Table S4D for details). Taken together, these data show that, similarly to Foxo, Aop functions downstream of *chico* and is essential for *chico-*dependent lifespan extension.

### Pharmacological Inhibition of Ras-Erk-ETS Signaling Extends Lifespan

The prolific role of Ras-Erk-ETS signaling in cancer has fueled an intense search for small-molecule inhibitors targeting this pathway. Trametinib, an FDA-approved drug for the treatment of melanoma, is one such potent and highly specific inhibitor of the Mek kinase, preventing activation of Erk by Ras (Yamaguchi et al., 2011).

**Figure 5:**
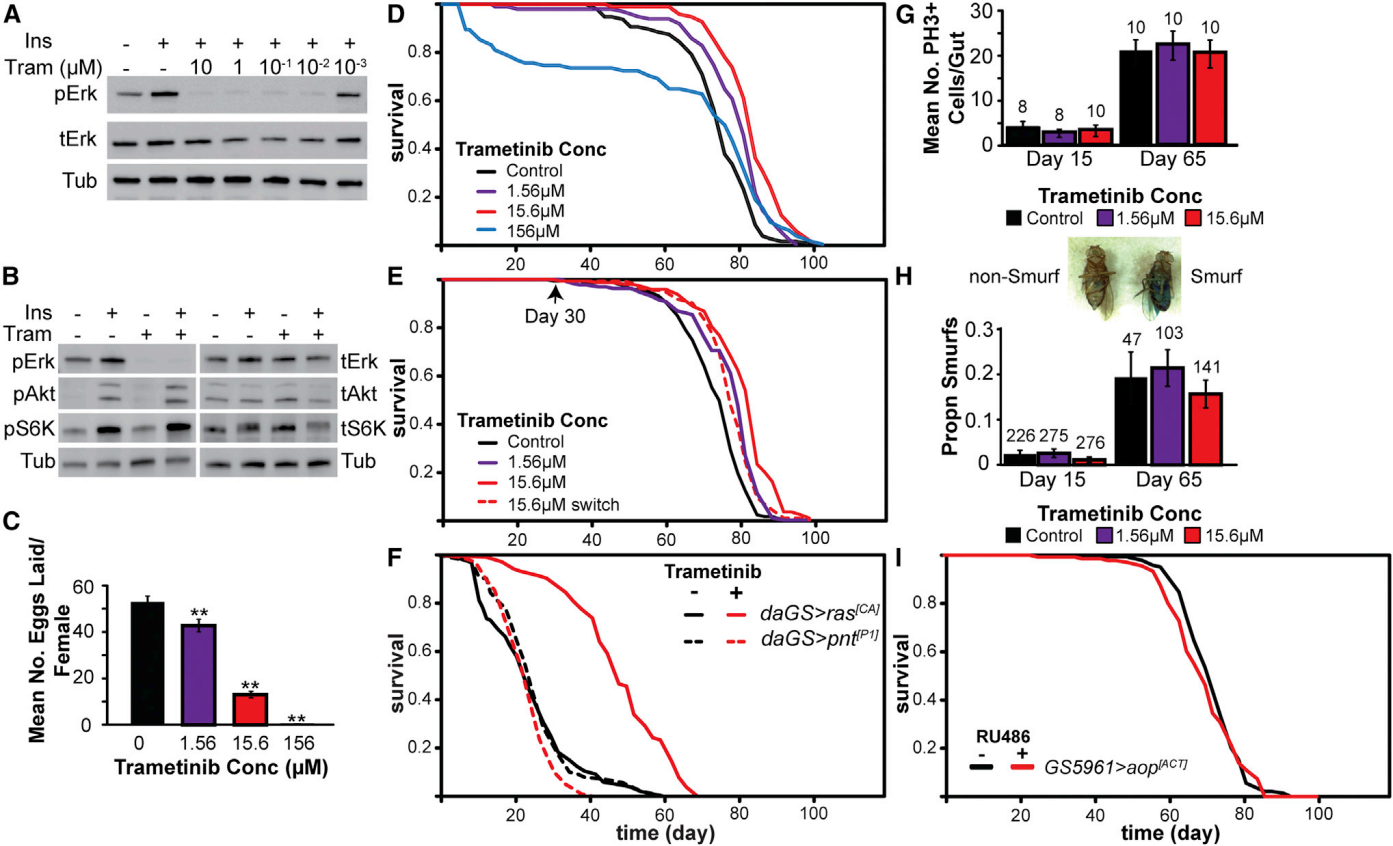
Pharmacological Inhibition of Ras-Erk Signaling Using Trametinib Extends Lifespan (A) *Drosophila* S2 cells treated with trametinib (Tram) at the indicated concentrations show inhibition of insulin-stimulated (Ins) Erk phosphorylation. (B) S2 cells treated with a high concentration of trametinib (10 μM) show no inhibition of insulin-dependent phosphorylation of Akt or S6K. (C) Effects of trametinib dose on female egg laying. Means ± SEM, ^∗∗^p < 0.05 t test (compared to 0 μM condition). (D) Trametinib extends lifespan in *wDah* females.(1.56 μM, p = 2.65 × 10^−4^; 15.6 μM, p = 1.92 × 10^−10^). See Table S5A. (E) Later-life (day 30) post-reproductive (see also Figure S5) exposure to 15.6 μM trametinib extends lifespan (p = 5.02 × 10^−5^). See Table S5B. (F) Trametinib increases survival of *daGS > ras*^*[CA]*^ (p = 1.24 × 10^−39^), but not *daGS > pnt*^*[P1]*^ flies (p = 0.59) in the presence of RU486. See Table S5C. (G) Age-related changes in gut ISC proliferation in animals exposed to trametinib for 15 or 65 days. Mean number of PH3+ cells per gut ± SEM. Number of guts analyzed are indicated above each bar. GLM with Poisson distribution and overdispersion parameter: significant effect of age (p < 0.001), but not trametinib concentration (p = 0.84) or their interaction (p = 0.79). (H) Age-related changes in intestinal integrity in animals exposed to trametinib for 15 or 65 days. Proportion of smurfs present in the population ± SE. Total numbers of flies examined for each condition are indicated above each bar. GLM with binomial distribution and overdispersion parameter: significant effect of age (p < 0.001), but not trametinib concentration (p = 0.84) or their interaction (p = 0.79). (I) No significant differences in survival of *GS5961 > aop*^*[ACT]*^ females in the presence or absence of RU486 (p = 0.45). See Table S5D.

We first confirmed that trametinib inhibits Erk activation in *Drosophila* S2 cells. Pre-treatment with trametinib over a wide range of concentrations completely blocked both basal and insulin-stimulated Erk activation (Figure 5A). Furthermore, cells treated with the highest dose of 10 μM trametinib did not show any inhibition of insulin-stimulated Akt or S6K phosphorylation (Figure 5B). Thus, trametinib is a potent and specific inhibitor of Ras-Erk signaling in flies.

To validate the Ras-Erk-ETS pathway as a drug target for extension of animal lifespan, we tested the effects of trametinib on *Drosophila* survival. Since loss-of-function mutants for *Drosophila ras* are female sterile (Rørth, 1996), we first monitored the effects of the drug on female egg laying in order to determine the biologically relevant doses (Figure 5C). The drug was administered to adult flies orally by supplementation in the food medium, and we selected the doses that produced small to essentially complete inhibition of egg production (1.56 μM to 156 μM) for lifespan assays. Based on quantifications of daily *Drosophila* food intake (Deshpande et al., 2014), we estimate that flies housed on food containing 1.56 μM trametinib ingest ∼0.4 mg/kg body weight of the drug per day, which is comparable to the oral dose used in mammalian studies of 1 mg/kg body weight (Yamaguchi et al., 2011) and the treatment dose of 2 mg per day for human cancer patients (Infante et al., 2012).

**Figure S3:**
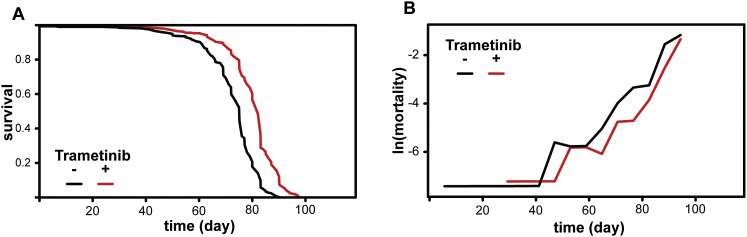
Mortality Analysis of Flies Exposed to 15.6 μM Trametinib, Related to Figure 5 (A) Combined survival data from three independent trials of *wDah* females exposed to 15.6 μM trametinib. Log-rank detected a significant difference in survival between trametinib-treated flies and untreated controls (increase in median lifespan of 9%, p < 0.0001). Controls: n = 338 deaths/8 censors, median lifespan = 75 days; trametinib-treated: n = 325/28, median lifespan = 82 days. (B) Age-specific mortality analysis of the survival data in (A). Mortality (μx) was calculated as: μx = -ln(1-qx), where qx (the probability of dying in a time interval) was averaged per day over a 5-day interval and is shown at the end of the interval. Intervals with zero deaths were removed. Estimates of the parameters of the Gompertz mortality model: λ (baseline mortality) controls = 7.3x10^−6^, trametinib-treated = 2.3x10^−6^, p = 0.045; γ (change in mortality with age) controls = 0.13, trametinib-treated = 0.13, p = 0.67.

**Figure S4:**
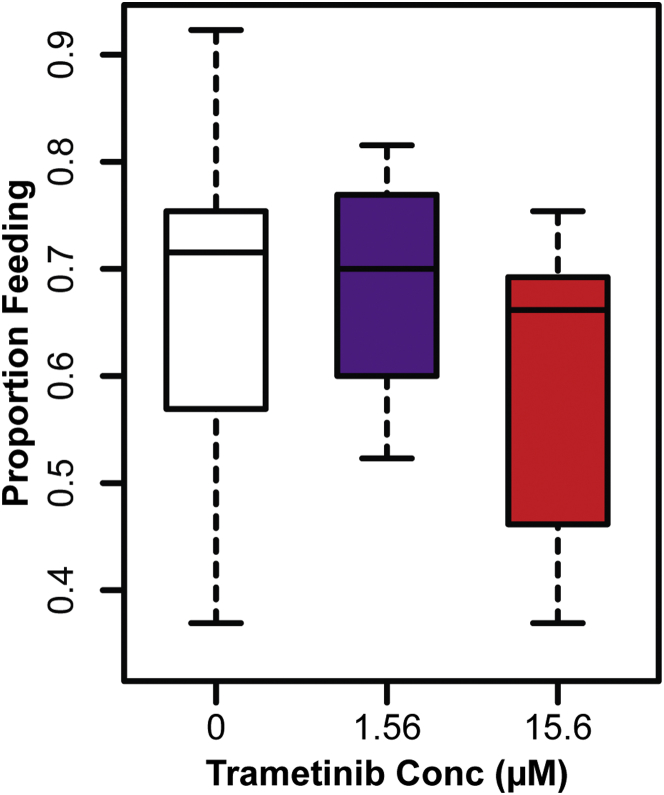
Trametinib Does Not Affect Feeding Behavior, Related to Figure 5 Feeding events as a proportion of total events observed per vial for *wDah* females on food containing 0 μM, 1.56 μM and 15.6 μM trametinib (15 vials per condition). Data were analyzed using a Generalized Linear Model with binomial distribution and overdispersion parameter. There was no significant effect of trametinib concentration on feeding (p = 0.66).

1.56 μM and 15.6 μM trametinib significantly increased fly lifespan (Figure 5D: 1.56 μM, median lifespan +8%, p = 2.65 × 10^−4^; 15.6 μM, median lifespan +12%, p = 1.92 × 10^−10^). CPH analysis revealed that, in this dose range, the risk of death per day was reduced by 4.7% per μM of trametinib (95% CI: 2.9%–6.3%, Table S5A). Mortality analysis showed that treatment with 15.6 μM trametinib reduced demographic frailty (baseline mortality) with no significant difference in the rate of change of mortality with age (Figure S3). Such a change in baseline mortality would be preferable for a pharmacological intervention for aging, since it would delay the onset of mortality without prolonging the duration of age-related decline. Higher trametinib concentrations (156 μM) resulted in early life mortality but improved survival later in life, resulting in significant increases in maximum, but not median, lifespan. Importantly, no significant effects of trametinib were observed on feeding behavior at doses conducive to lifespan extension (Figure S4).

**Figure S5:**
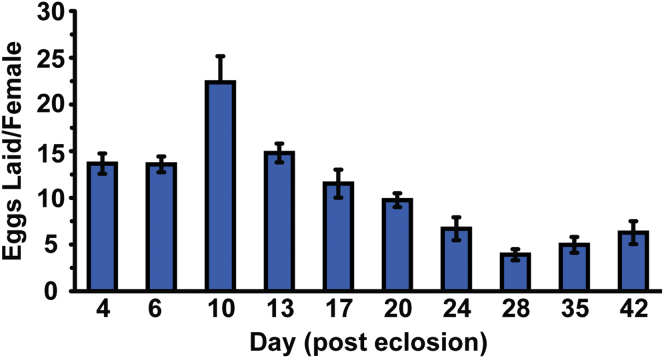
Egg Laying Over Time in *wDah* Females, Related to Figure 5 Egg-laying in *wDah* females peaks at around day 10 post-mating and then declines to plateau by day 28. Means ± SEM.

For any pharmacological intervention into aging, it is important it be beneficial even when started late in life. We therefore administered the drug to female flies from 30 days post-eclosion, when egg laying has almost ceased (Figure S5). This late administration also resulted in a significant extension of lifespan (Figure 5E and Table S5B, median lifespan + 4%, p = 5.02 × 10^−5^). Continuous exposure to the drug resulted in bigger effects on lifespan than when drug administration was restricted to later life, possibly reflecting cumulative effects of earlier drug exposure.

**Figure S6:**
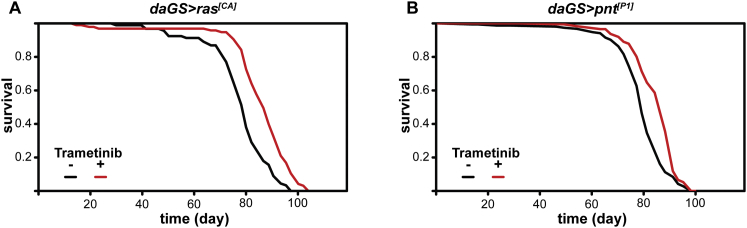
Trametinib Extends the Lifespan of *daGS > ras*^*[CA]*^ and *daGS > pnt*^*[P1]*^ Flies in the Absence of Transgene Expression, Related to Figure 5 (A and B) In the absence of RU486 and hence transgene expression, trametinib treatment at 15.6 μM increased the survival of *daGS > ras*^*[CA]*^ flies (increase in median lifespan of 9%, p = 2.07x10^−8^) and *daGS > pnt*^*[P1]*^ flies (increase in median lifespan of 9%, p = 2.69x10^−7^). Untreated *daGS > ras*^*[CA]*^: n = 90 deaths/6 censors, median lifespan = 82 days; trametinib-treated *daGS > ras*^*[CA]*^: n = 95/1, median lifespan = 89 days; untreated *daGS/ pnt*^*[P1]*^, n = 141/6, median lifespan = 79 days; trametinib-treated daGS*> pnt*^*[P1]*^, n = 132/19, median lifespan = 86 days.

To confirm that trametinib extends lifespan by inhibition of Ras-Erk-ETS signaling, we examined the effects of trametinib on the survival of flies expressing constitutively active Ras or constitutively active Pnt. In the presence of RU486, trametinib treatment at 15.6 μM doubled the lifespan of *daGS > ras*^*[CA]*^ (Figure 5F, p = 1.24 × 10^−39^), while the survival of *daGS > pnt*^*[P1]*^ was unaffected by drug treatment (Figure 5F, p = 0.59). CPH analysis confirmed that trametinib had a significantly different impact on the lifespan of *daGS > ras*^*[CA]*^ flies compared to *daGS > pnt*^*[P1]*^ flies in the presence RU486 (Table S5C, p < 2 × 10^−16^). In the absence of transgene expression, 15.6 μM trametinib treatment significantly increased the lifespan of both genotypes (Figure S6). Hence, trametinib acts specifically between Ras and Pnt to extend fly lifespan. In summary, our data confirm that the Ras-Erk-ETS pathway is a valid drug target for extension of animal lifespan.

### Trametinib Does Not Act on Lifespan via Modulation of Stem Cell Proliferation

Inhibition of Ras-Erk signaling can have substantial effects on cell proliferation. Adult *Drosophila* somatic tissues are predominantly post-mitotic, although a population of adult stem cells resides in the midgut, and their proliferative capacity, in part regulated by Ras-Erk signaling, is important for lifespan (Biteau and Jasper, 2011; Biteau et al., 2010). To test whether trametinib affects age-dependent intestinal stem cell (ISC) proliferation rates, we determined the frequency of phospho-histone H3-positive (pH3+) cells, a marker of cell-cycle progression and a direct measurement of ISC proliferation (Biteau et al., 2010). The number of PH3+ cells increased 5-fold from 15 to 65 days in control flies, and continuous exposure to 1.56 μM or 15.6 μM trametinib did not prevent this age-dependent increase in ISC proliferation (Figure 5G). Hence, trametinib does not significantly affect stem cell proliferation at doses that extend lifespan.

To further confirm that gut function was unaffected by trametinib, we examined gut intestinal barrier function, which is a good indicator of overall intestinal integrity and is important for survival (Rera et al., 2012). Flies were fed a non-absorbable blue food dye, and “smurf” flies, in which the gut is unable to prevent this dye from leaking into the hemolymph, scored (Rera et al., 2012). The proportion of smurfs in the control population increased by 8-fold from 15 to 65 days (Figure 5H). Again, trametinib did not alter this age-dependent loss of intestinal epithelial integrity (Figure 5H). Consistent with these observations, we found that adult-onset expression of Aop^[ACT]^ specifically within the ISCs is not sufficient to extend lifespan (Figure 5I and Table S5D, p = 0.45). Hence, the effects of pharmacological or genetic manipulation of Ras-Erk-ETS signaling cannot be explained by the modulation of ISC proliferation and maintenance of gut function.

## Discussion

### Ras-Erk-ETS Signaling as an Effector of the IIS Longevity Response

The key role of IIS in determining animal lifespan has been well appreciated for more than two decades and shows strong evolutionary conservation (Fontana et al., 2010; Kenyon, 2011). Alleles of genes encoding components of this pathway have also been linked to longevity in humans (Bonafè et al., 2003; Kuningas et al., 2007; Suh et al., 2008). Multiple studies have demonstrated the importance of the PI3K-Akt-Foxo branch of IIS, while in this study we identify an equally important role for Ras-Erk-ETS signaling in IIS-dependent lifespan extension.

We have shown that, downstream of *chico*, preventing the activation of either Ras or PI3K is sufficient to extend lifespan. Ras can interact directly with the catalytic subunit of PI3K, which is required for maximal PI3K activation during growth (Orme et al., 2006). Thus, inhibition of Ras could increase lifespan via inactivation of PI3K. However, several lines of evidence indicate that the Erk-ETS pathway must also, if not solely, be involved. In this study and elsewhere, we demonstrated that direct inhibition of the Ras-dependent kinase, Erk, or activation of the Aop transcription factor, a negative effector of the Ras-Erk pathway, is sufficient to extend lifespan. Importantly, we show that Ras-Erk-ETS signaling is genetically linked to *chico* because activation of Aop is required for lifespan extension due to *chico* loss of function. Furthermore, altering the ability of Chico to activate Ras or PI3K does not result in equivalent phenotypes: we and others (Oldham et al., 2002) showed that mutation of the Grb2/Drk docking site in Chico is dispensable for multiple developmental phenotypes associated with *chico* mutation, while disruption of the Chico-PI3K interaction is not. Overall, our observations strongly suggest that lifespan extension downstream of *chico* mutation involves inhibition of the Ras-Erk-ETS-signaling pathway.

**Figure 6:**
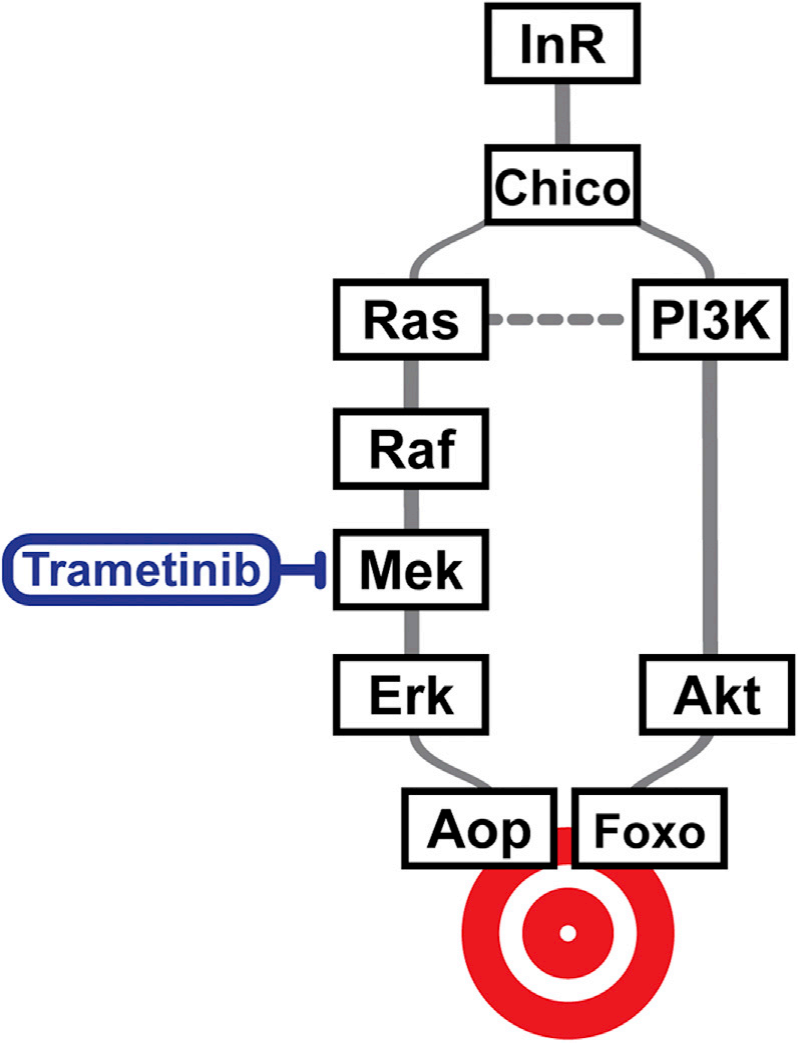
Model of Aop-Foxo Function Downstream of IIS We propose that, downstream of the insulin receptor substrate, Chico, signaling via the IIS pathway bifurcates into two branches: Ras-Erk and PI3K-Akt. At the transcriptional level, these two branches subsequently re-join, acting on the Aop and Foxo TFs in a non-additive manner. The two TFs then co-operatively regulate the expression of a subset of target genes required for lifespan extension.

The simplest model to integrate the role of Ras-Erk-ETS signaling with the PI3K-Akt-Foxo branch in extension of lifespan by reduced IIS is presented in Figure 6. We propose that, downstream of Chico, the IIS pathway bifurcates into branches delineated by Erk and Akt, with inhibition of either sufficient to extend lifespan, as is activation of either responsive TF, Aop or Foxo. The two branches are not redundant, because mutation of *chico* or the loss of its ability to activate either branch results in the same magnitude of lifespan extension. Furthermore, Aop and Foxo are each individually required downstream of *chico* mutation for lifespan extension. At the same time, the effects of the two branches are not additive, as simultaneous activation of Aop and Foxo does not extend lifespan more than activation of either TF alone (Alic et al., 2014). Taken together, these data suggest that the two pathways re-join for transcriptional regulation, where Aop and Foxo co-operatively regulate genes required for lifespan extension. Our model is corroborated by our previous finding that, in the adult gut and fat body, some 60% of genomic locations bound by Foxo overlap with regions of activated-Aop binding (Alic et al., 2014). We propose that functional interactions of Aop and Foxo at these sites may be such that each factor is both necessary and sufficient to achieve the beneficial changes in target gene expression upon reduced IIS.

It remains to be determined how promoter-based Foxo and Aop interactions produce such physiologically relevant, transcriptional changes. It is, however, curious that activation of either TF alone promotes longevity when one is known as a transcriptional activator (Foxo) and the other as a transcriptional repressor (Aop). We have consistently observed a subset of Foxo-bound genes, albeit a minority, that are transcriptionally repressed when Foxo is activated (Alic et al., 2011, 2014). Furthermore, the Foxo target gene *myc* is downregulated in larval muscle when Foxo is active under low insulin conditions, while deletion of *foxo* or its binding site within the *myc* promoter results in de-repression of myc expression in adipose of fed larvae (Teleman et al., 2008). Thus, on some promoters under certain conditions, *Drosophila* Foxo appears to act as a transcriptional repressor. Mammalian Foxo3a may also directly repress some genes (Wu et al., 2013; Yang et al., 2014). It will therefore be important to test whether the lifespan-relevant interactions between Foxo and Aop occur on promoters where Foxo acts as a repressor with Foxo possibly acting as a cofactor for Aop or vice versa.

In mediating the effects of IIS on lifespan, the Ras-Erk-ETS- and PI3K-Akt-Foxo-signaling pathways both appear to inhibit Aop/Foxo. To understand why signaling might be so wired, it is important to consider that the two pathways are also regulated by other stimuli, such as other growth factors, stress signals, and nutritional cues. The re-joining of the two branches at the transcriptional level would therefore allow for their outputs to be integrated, producing a concerted transcriptional response, a feature that is also seen in other contexts. For example, stability of the Myc transcription factor is differentially regulated in response to Erk and PI3K signals, allowing it to integrate signals from the two kinases (Lee et al., 2008). Transcriptional integration in response to RTK signaling also confers specificity during cell differentiation, with combinatorial effects of multiple transcriptional modulators inducing tissue-specific responses to inductive Ras signals (Halfon et al., 2000). Similar integrated responses of lifespan could be orchestrated by transcriptional coordination of Aop and Foxo.

### A Role for Ras-Erk-ETS Signaling in Mammalian Aging

We find that direct inhibition of Ras in *Drosophila* can extend lifespan, suggesting that the role of Ras in aging is evolutionarily conserved. In budding yeast, deletion of *RAS1* extends replicative lifespan (Sun et al., 1994), and deletion of *RAS2* increases chronological lifespan by altering signaling through cyclic-AMP/protein kinase A (cAMP/PKA) (Fabrizio et al., 2003), downregulation of which is sufficient to extend both replicative and chronological lifespan (Fabrizio et al., 2001, 2004; Lin et al., 2000). This role of cAMP/PKA in aging may be conserved in mammals, as disruption of adenylyl cyclase 5′ and PKA function extend murine lifespan (Enns et al., 2009; Yan et al., 2007). However, cAMP/PKA are not generally considered mediators of Ras function in metazoa. Instead, our data suggest that signaling through Erk and the ETS TFs mediates the longevity response to Ras. Interestingly, fibroblasts isolated from long-lived mutant strains of mice and long-lived species of mammals and birds show altered dynamics of Erk phosphorylation in response to stress (Elbourkadi et al., 2014; Sun et al., 2009), further suggesting a link between Erk activity and longevity. Importantly, the ETS TFs are conserved mediators of Ras-Erk signaling in mammals (Sharrocks, 2001). Investigation of the effects of Ras inhibition on mammalian lifespan and the role of the mammalian Aop ortholog Etv6 are now warranted.

### Ras-Erk-ETS Inhibitors as Potential Anti-Aging Therapeutics

A role for Ras-Erk-ETS signaling in lifespan offers multiple potential targets for small-molecule inhibitors that could function as anti-aging interventions. Importantly, due to the key role of this pathway in cancer, multiple such inhibitors exist or are in development (Neuzillet et al., 2014).

We have shown that trametinib, a highly specific allosteric inhibitor of the Mek kinase (Yamaguchi et al., 2011), prolongs *Drosophila* lifespan, thus validating the Ras-Erk-ETS pathway as a pharmacological target for anti-aging therapeutics. Trametinib joins a very exclusive list of FDA-approved drugs that promote longevity in animals (de Cabo et al., 2014), the most convincing other example being rapamycin.

Rapamycin not only increases lifespan in multiple organisms, including mammals, but also improves several indices of function during aging (Ehninger et al., 2014; Lamming et al., 2013). While rapamycin can protect against tumor growth (Anisimov et al., 2011), the effects on longevity appear to be independent of cancer prevention, as rapamcyin-treated animals still develop tumors (Harrison et al., 2009) and rapamycin can increase lifespan in tumor-free species (Bjedov et al., 2010). Furthermore, increased activity of certain tumor suppressors such as lnk4a/Arf and PTEN as well as the *RasGrf1* deficiency all increase lifespan independently of anti-tumor activity (Borrás et al., 2011; Matheu et al., 2009; Ortega-Molina et al., 2012). Our findings that trametinib can increase lifespan in *Drosophila*, which are mainly post-mitotic in adulthood, and that doses of trametinib that increase lifespan do not alter proliferation rates of ISCs in *Drosophila* suggest that the anti-aging effects of trametinib are separable from its anti-cancer activity.

Finally, due to the high degree of evolutionary conservation in the Ras-Erk-ETS pathway, our study suggests the intriguing possibility that pharmacological inhibition of Ras-Erk-ETS may also increase lifespan in mammals.

## Experimental Procedures

### Fly Stocks and Husbandry

Stocks were maintained and experiments conducted at 25°C on a 12 hr:12 hr light:dark cycle at 60% humidity, on food containing 10% (w/v) brewer’s yeast, 5% (w/v) sucrose, and 1.5% (w/v) agar unless otherwise noted. RU486 (Sigma) dissolved in ethanol was added to a final concentration of 200 μM. Trametinib (LC Laboratories) was added from a 62.4 mM stock solution in DMSO maintaining a final DMSO concentration of 0.25% (v/v). For control treatments, equivalent volumes of the vehicle alone were added. Stocks were backcrossed for at least six generations into the wild-type outbred *wDahomey* population, with the exception of *UAS-ras85D*^*[RNAi]*^ and *UAS-rl*^*[RNAi]*^, which were used as hybrids. *Drosophila* stocks, cloning strategies, and phenotyping are described in the Supplemental Experimental Procedures. For lifespans, flies were sorted into experimental vials at a density of 10 or 15 flies per vial. Flies were transferred to fresh vials three times a week, and deaths/censors were scored during transferral.

### Bimolecular Fluorescence Complementation Assays

Constructs encoding the wild-type Chico protein or Chico Grb2/Drk-binding site mutant and *Drosophila* Drk were transfected into S2 cells. After 3 days, cells were serum starved for 2 hr and then stimulated with 1 μM human insulin (Sigma) for 24 hr before imaging.

### Immunofluorescence

Aop immunofluorescence was performed on dissected adult abdominal fat bodies using a monoclonal mouse anti-AOP antibody (Rebay and Rubin, 1995) at 1:100 dilution. Phospho-histone H3 immunofluorescence was performed on dissected adult midguts using a polyclonal rabbit phospho-histone H3 (Ser10) antibody (Cell Signaling).

### Western Blots

S2 cells were serum starved for 2 hr, treated with trametinib dissolved in DMSO or DMSO alone for 30 min, and then stimulated with 1 μM human insulin (Sigma) for 15 min. Western blots were probed for phospho-Erk(Thr202/Tyr204) (#4370), phospho-Akt(Ser473) (#4060), phospho-S6K(Thr398) (#9209), total-Erk (#4695), total-Akt (#9272) (Cell Signaling Technologies), total-S6K, and tubulin.

### qRT-PCR Analysis

Total RNA was isolated from either five whole adult flies or five dissected abdominal fat bodies using standard TRIZOL (Invitrogen) protocols and converted to cDNA using oligod(T) primer and Superscript II reverse transcriptase (Invitrogen). Quantitative RT-PCR was performed using Power SYBR Green PCR Master Mix (ABI), and relative quantities of transcripts were determined using the relative standard curve method normalized to *actin5C*. See Supplemental Experimental Procedures for primer sequences.

### Statistical Analysis

Statistical analysis was performed in Excel (Microsoft), Jmp version 9 (SAS Insitute), or R, except for mortality analysis in which survival data were fitted to the Gompertz model using Survomatic online. Statistical tests used are indicated in the figure captions. See the Supplemental Experimental Procedures for further details.

## Author Contributions

L.P. conceived and designed the study. C.S. and N.A. designed the study, co-ordinated experiments, and did the experimental work along with A.F., M.C., and M.P.H. C.S. demonstrated that Ras is involved in IIS-dependent lifespan extension and that pharmacological inhibition of Ras-Erk-ETS signaling extends lifespan. N.A. made the initial discovery that inhibition of Ras extends lifespan and showed that it acts through Aop. C.S., N.A., and L.P. drafted the manuscript.

## Supplemental Information

Document S1. Supplemental Experimental Procedures

Table S1. Ras Inhibition Is Required Downstream of IIS to Extend Lifespan, Related to Figure 1 Statistical analysis of survival data presented in Figure 1.

Table S2. Direct Inhibition of Ras-Erk Signaling Extends Lifespan, Related to Figure 2 Statistical analysis of survival data presented in Figure 2.

Table S3. *aop* Functions Downstream of Ras in the Adult Fly to Control Lifespan Statistical analysis of survival data presented in Figure 3.

Table S4. *aop* Is Required Downstream of *chico* to Extend Lifespan Statistical analysis of survival data presented in Figure 4.

Table S5. Trametinib Extends *Drosophila* Lifespan Statistical analysis of survival data presented in Figure 5.

Document S2. Article plus Supplemental Information
