# Supplementary figures and images for: The Ras-Erk-ETS-Signaling Pathway Is a Drug Target for Longevity
Source: Cell. 2015 Jul 2;162(1):72–83. doi: 10.1016/j.cell.2015.06.023 (PMC4518474; doi:10.1016/j.cell.2015.06.023)

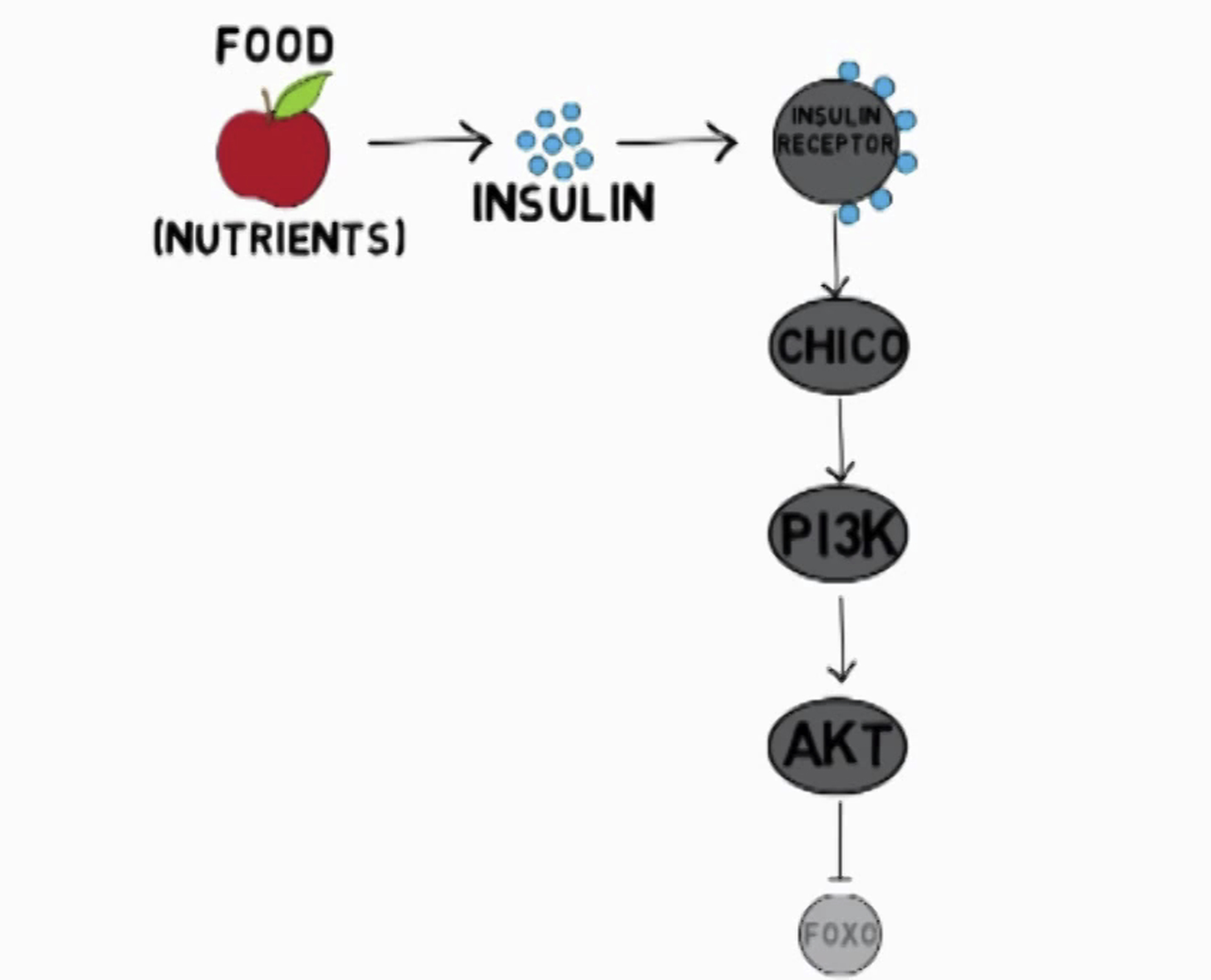

Supplement: Supplementary file 1 [file mmc8.jpg]
