# Supplementary material for: The Ras-Erk-ETS-Signaling Pathway Is a Drug Target for Longevity
Source: Cell. 2015 Jul 2;162(1):72–83. doi: 10.1016/j.cell.2015.06.023 (PMC4518474; doi:10.1016/j.cell.2015.06.023)
Supplement: Document S1. Supplemental Experimental Procedures [file mmc1.pdf]

Cell

Supplemental Information

The Ras-Erk-ETS-Signaling Pathway Is a Drug Target for Longevity

Cathy Slack, Nazif Alic, Andrea Foley, Melissa Cabecinha, Matthew P. Hoddinott, and Linda Partridge

Supplemental Experimental Procedures

Fly Stocks and Culture

The following fly stocks were all obtained from the Bloomington Stock Centre: *chico*^{1} (Bohni et al., 1999), *UAS-aop*$^{[ACT]}$ (Rebay and Rubin, 1995), *UAS-pnt*$^{[P1]}$ (O'Neill et al., 1994), *UAS-ras*$^{[N17]}$ (referred to as *UAS-ras*$^{[DN]}$) (Lee et al., 1996) and *UAS-ras*$^{[V12]}$ (referred to as *UAS-ras*$^{[CA]}$). *UAS-ras85D*$^{[RNAi]}$ (HMS01294), *UAS-rl*$^{[RNAi]}$ (HMS00173) and *UAS-aop*$^{[RNAi]}$ (HMS01256) were from the TRiP collection. *daGS* was a gift from Veronique Monnier (Tricoire et al., 2009) and *GS5961* was kindly provided by Benjamin Ohlstein (Mathur et al., 2010). Other stocks used in this study are *actGS* (Poirier et al., 2008), $S_{1}106$ (Giannakou et al., 2004) and *dfoxoΔ94* (Slack et al., 2011). A 5.6 kb genomic fragment including the entire *chico* locus was used to generate the *chico* genomic rescue construct. Point mutations were introduced using the QuikChange II Site-Directed Mutagenesis Kit (Agilent Technologies). All genomic rescue constructs were inserted into the genome by means of ΦC31 mediated integration into a landing site on the third chromosome at 68A2. The wild-type stock *Dahomey* was collected in 1970 in Dahomey (now Benin) and has since been maintained in large population cages with overlapping generations on a 12L:12D cycle at 25 °C. *w Dahomey* was derived from backcrossing the *w*^{1118} mutation into *Dahomey*.

For all experiments, flies were maintained on standard sugar/yeast/agar media (Bass et al., 2007). Flies were reared at standard larval density and eclosing adults were collected over a 12 hour period. Flies were mated for 48 hours before females were sorted onto different food conditions.

Developmental Phenotyping

Developmental time was measured by collecting eggs over a period of 3 hours. Embryos were allowed to hatch and first instar larvae were hand-picked and transferred (50 per vial)

on to standard food. When adult flies started to hatch the number of eclosed individuals was counted at regular intervals. For body weight determination, flies were briefly anaesthetized on ice and weighed individually on a ME235S analysis balance (Sartorius Mechantronics). For female fecundity tests, female flies were housed with males for 48 hours post-eclosion and then separated into vials at a density 10 females per vial. Eggs were collected over a 24-hour period on day 7 post-eclosion and the number of eggs laid per vial was counted. Gycogen and triglyceride levels in 7-day old adult females were measured as previously described (Slack et al., 2010).

Feeding Assay

Feeding rates were measured using a proboscis-extension assay in undisturbed conditions as previously described (Wong et al., 2009). Trametinib-treated flies were assayed after 7 days of exposure to the drug. Flies were housed at a density of 5 flies per vial and transferred to new food on the evening before the assay. Feeding data is expressed as a proportion by experimental group (sum of scored feeding events divided by total number of feeding opportunities, where total number of feeding opportunities = number of flies in vial×number of vials in the group×number of observations). For statistical analyses, comparisons between experimental groups were made on the totals of feeding events by all flies within a vial, to avoid pseudoreplication.

Smurf Assay

Flies were aged on standard food until the day before the assay. Flies were examined for the "smurf" phenotype using 2.5% (w/v) FD&C blue dye no. 1 (Fastcolours) as previously described (Rera et al., 2012), except the flies were kept on the blue food for 18 hours.

BiFC Cloning and Expression

chico genomic sequences encoding the full-length wild-type Chico protein sequence, the Grb2/Drk-binding mutant and the *Drosophila drk* open reading frame were cloned into pUAST-BiFC plasmids (a gift from S. Bogdan; (Gohl et al., 2010)) using standard Gateway cloning techniques (Invitrogen). Constructs were cotransfected into S2 cells with pAct-GAL4 to induce expression.

Imaging and Quantification

For BiFC assays and phospho histone H3 labelled cells, fluorescence images were acquired and counted using a Zeiss Axioskop2 plus microscope using a GFP filter cube (excitation: 470/40 nm; emission: 525/50 nm), an ORCA-ER digital camera (Hamamatsu) and Volocity image analysis software (Improvision). For nuclear AOP staining, images were captured using the Zeiss LSM 700 confocal microscope and quantified using ImageJ in at least 3 nuclei per animal, for 5 animals per treatment group. Images were processed for presentation (brightness/contrast/colour adjusted) in Photoshop CS6.

Western Blots

After insulin stimulation, trametinib-treated S2 cells were lysed in Laemmli buffer containing 0.1 mM DTT. Proteins were resolved on 4-15% Tris-Glycine-SDS gradient gels (Biorad) and transferred to nitrocellulose membranes. The total S6K antibody was raised in rabbits against a previously reported peptide sequence (Stewart et al., 1996). Secondary antibodies conjugated to HRP (Abcam) were used at a dilution of 1:10000 and signals detected using the Luminata Forte chemiluminescent HRP substrate (Millipore).

Primer sequences for qRT-PCR

chico

forward: ATCAGGCGATGCGGTC

reverse: ACATAGCGCTCAGTATCG

la costa

forward: GCTGTGGTGGTGGAAGTGCT

reverse: GGACCTCCATCCAACCAAAC

CG1678

forward: CGGACGCCTCACTCGGAG

reverse: CCCAAGGTGGCAACTCATC

actin5C:

forward: CACACCAAATCTTACAAAATGTGTGA

reverse: AATCCGGCCTTGCACATG

Statistical Analysis

Survival data were analysed with either Log-rank test or Cox Proportional Hazard using Excel (Microsoft), Jmp version 9 (SAS Insitute) or R using *survival* package (Terry Therneau, http://CRAN.R-project.org/package=survival). For mortality analysis, survival data were fitted to the Gompertz model and significant differences in the model parameters λ (baseline mortality) and γ (change in mortality with age) between treatments were determined using Survomatic online (http://spark.rstudio.com/bokov/sm/) developed by Alex Bokov and based on (Pletcher, 1999). BiFC YFP fluorescence data was analysed using Chi squared in Excel. Aop nuclear localisation data were analysed with t-test in JMP. mRNA expression data were log transformed and analysed with a mixed effects linear model in JMP with dissection/batch as a random effect followed by post-hoc, pair-wise t-test (where indicated).

Age-related changes in PH3+ cell number were analysed using a Generalised Linear Model with poisson distribution and overdispersion parameter in Jmp. Smurf data were analysed using a Generalised Linear Model with binomial distribution and overdispersion parameter in Jmp. One-way analyses of variance (ANOVA) were performed and planned comparisons of means were made using Tukey-Kramer HSD or Students t-test in Jmp.

Supplemental References

Bass, T.M., Grandison, R.C., Wong, R., Martinez, P., Partridge, L., and Piper, M.D. (2007). Optimization of dietary restriction protocols in Drosophila. The journals of gerontology Series A, Biological sciences and medical sciences *62*, 1071-1081.

Bohni, R., Riesgo-Escovar, J., Oldham, S., Brogiolo, W., Stocker, H., Andruss, B.F., Beckingham, K., and Hafen, E. (1999). Autonomous control of cell and organ size by CHICO, a Drosophila homolog of vertebrate IRS1-4. Cell *97*, 865-875.

Giannakou, M.E., Goss, M., Junger, M.A., Hafen, E., Leevers, S.J., and Partridge, L. (2004). Long-lived Drosophila with overexpressed dFOXO in adult fat body. Science *305*, 361.

Gohl, C., Banovic, D., Grevelhorster, A., and Bogdan, S. (2010). WAVE forms hetero- and homo-oligomeric complexes at integrin junctions in Drosophila visualized by bimolecular fluorescence complementation. The Journal of biological chemistry *285*, 40171-40179.

Lee, T., Feig, L., and Montell, D.J. (1996). Two distinct roles for Ras in a developmentally regulated cell migration. Development *122*, 409-418.

Mathur, D., Bost, A., Driver, I., and Ohlstein, B. (2010). A transient niche regulates the specification of Drosophila intestinal stem cells. Science *327*, 210-213.

O'Neill, E.M., Rebay, I., Tjian, R., and Rubin, G.M. (1994). The activities of two Ets-related transcription factors required for Drosophila eye development are modulated by the Ras/MAPK pathway. Cell *78*, 137-147.

Pletcher, S.D. (1999). Model fitting and hypothesis testing for age-specific mortality data. Journal of Evolutionary Biology *12*, 430-439.

Poirier, L., Shane, A., Zheng, J., and Seroude, L. (2008). Characterization of the Drosophila gene-switch system in aging studies: a cautionary tale. Aging Cell *7*, 758-770.

Rebay, I., and Rubin, G.M. (1995). Yan functions as a general inhibitor of differentiation and is negatively regulated by activation of the Ras1/MAPK pathway. Cell *81*, 857-866.

Rera, M., Clark, R.I., and Walker, D.W. (2012). Intestinal barrier dysfunction links metabolic and inflammatory markers of aging to death in Drosophila. Proceedings of the National Academy of Sciences of the United States of America *109*, 21528-21533.

Slack, C., Giannakou, M.E., Foley, A., Goss, M., and Partridge, L. (2011). dFOXO-independent effects of reduced insulin-like signaling in Drosophila. Aging Cell *10*, 735-748.

Slack, C., Werz, C., Wieser, D., Alic, N., Foley, A., Stocker, H., Withers, D.J., Thornton, J.M., Hafen, E., and Partridge, L. (2010). Regulation of lifespan, metabolism, and stress responses by the Drosophila SH2B protein, Lnk. PLoS genetics *6*, e1000881.

Stewart, M.J., Berry, C.O., Zilberman, F., Thomas, G., and Kozma, S.C. (1996). The Drosophila p70s6k homolog exhibits conserved regulatory elements and rapamycin sensitivity. Proceedings of the National Academy of Sciences of the United States of America *93*, 10791-10796.

Tricoire, H., Battisti, V., Trannoy, S., Lasbleiz, C., Pret, A.M., and Monnier, V. (2009). The steroid hormone receptor EcR finely modulates Drosophila lifespan during adulthood in a sex-specific manner. Mechanisms of ageing and development *130*, 547-552.

Wong, R., Piper, M.D., Wertheim, B., and Partridge, L. (2009). Quantification of food intake in Drosophila. PloS one *4*, e6063.
